# Bayesian Modeling of the Dynamics of Phase Modulations and their Application to Auditory Event Related Potentials at Different Loudness Scales

**DOI:** 10.3389/fncom.2016.00002

**Published:** 2016-01-28

**Authors:** Zeinab Mortezapouraghdam, Robert C. Wilson, Lars Schwabe, Daniel J. Strauss

**Affiliations:** ^1^Systems Neuroscience and Neurotechnology Unit, Faculty of Medicine, Saarland University, Homburg/Saar and School of Engineering, Saarland University of Applied SciencesSaarbrücken, Germany; ^2^Mathematical Image Processing and Data Analysis Group, Department of Mathematics, Technical University KaiserslauternKaiserslautern, Germany; ^3^Department of Psychology and Cognitive Science Program, University of ArizonaTucson, AZ, USA; ^4^Adaptive and Regenerative Software Systems, Department of Computer Science and Electrical Engineering, University of RostockRostock, Germany; ^5^Neurocognitive Haptics Lab at INM - Leibniz Institute for New MaterialsSaarbrücken, Germany; ^6^Key Numerics - Neurocognitive TechnologiesSaarbrücken, Germany

**Keywords:** Bayesian models, long-term habituation, instantaneous phase, event-related potentials, circular statistics

## Abstract

We study the effect of long-term habituation signatures of auditory selective attention reflected in the instantaneous phase information of the auditory event-related potentials (ERPs) at four distinct stimuli levels of 60, 70, 80, and 90 dB SPL. The analysis is based on the single-trial level. The effect of habituation can be observed in terms of the changes (jitter) in the instantaneous phase information of ERPs. In particular, the absence of habituation is correlated with a consistently high phase synchronization over ERP trials. We estimate the changes in phase concentration over trials using a Bayesian approach, in which the phase is modeled as being drawn from a von Mises distribution with a concentration parameter which varies smoothly over trials. The smoothness assumption reflects the fact that habituation is a gradual process. We differentiate between different stimuli based on the relative changes and absolute values of the estimated concentration parameter using the proposed Bayesian model.

## 1. Introduction

In this section we describe: (1) Definition of the habituation-mechanism, (2) The importance of analyzing phase information of auditory event related potentials (ERPs), and (3) Methods for objectively characterizing such phase information.

In almost all natural settings, animals are exposed to multiple concurrent streams of sensory information (Rosen, 1992; Chandrasekaran et al., 2010). To make sense of this sensory overload, it is crucial to filter it by increasing attention to stimuli that are important or novel and by decreasing attention to those that are irrelevant. One way of filtering out irrelevant information is through habituation, a simple form of learning that reflects a decrease in attention to repeated stimuli not caused by sensory adaptation or sensory fatigue (Rankin et al., 2009; Thompson, 2009; Domjan, 2014).

One neural signature of habituation can be found in the N100 wave component of auditory event related potentials (ERPs). ERPs are the endogenous measure of brain responses as a direct result of a specific sensory event. The non-invasive means of assessing habituation with ERPs has a number of clinical applications, from calibrating cochlear implants (especially with non-cooperative patients such as children) (Smoorenburg et al., 2002) to understanding pathological attentional binding in migraines, schizophrenia and tinnitus (Schoenen, 1997; Ludewig et al., 2002; Walpurger et al., 2003). The N100 component of the ERPs is defined in the latency period between 80 and 120 ms after the onset of stimuli. The link between the N100 wave and selective attention has been described in Hillyard et al. (1973). Habituation is hence believed to be explained as the gradual reduction in the selective attention to the stimulus which is reflected as a reduction of the N100 component of measured ERPs (Thompson, 2009). The reduction of the N100 wave can be observed both in the amplitude (Butler et al., 1969; Öhman and Lader, 1972; Hillyard et al., 1973; Rosburg et al., 2006) and phase (Babiloni et al., 2002; Busch et al., 2009; Low and Strauss, 2011; Mortezapouraghdam et al., 2014) of the signal.

**Figure 1:**
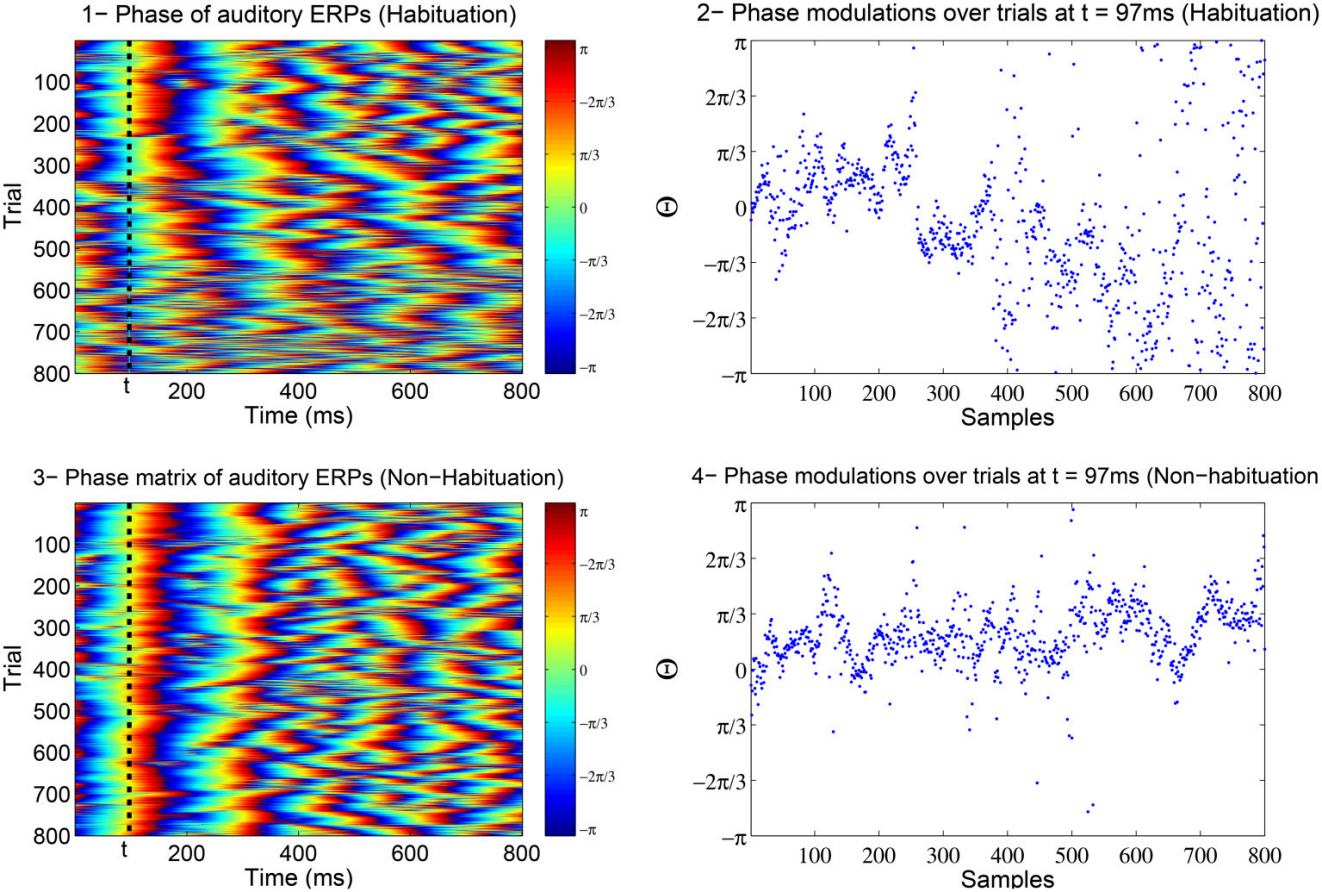
**The above example is the phase matrix of the N100 attenton-corelate wave of post-stimulus responses induced by a soft (number 1) and an aversive (number 3) stimuli**. The axis labeled as trials, correspond to the extracted phase information of every measured post-stimulus evoked response. The plots in 2 and 4 show the modulations of phase information at *t* = 97*ms* across the trials (responses). In case of a soft stimulus, the phase information are highly synchronized at the beginning of the experiment (high binding of the attention to stimulus) and then diffuse at the end of the experiment (attention drifts away). In case of an aversive stimulus, the phase information tend to stay highly synchronized throughout the experiment (high attention binding to stimulus).

In Low and Strauss (2011), the correlate of attention states in the averaged auditory ERP was detected to be reflected in the jitter of the instantaneous phase of subsequent single-trial ERPs (see Fell et al., 2004; Sauseng et al., 2007; Barry, 2009 for the relation between amplitude and phase). In Strauss et al. (2008) and Low and Strauss (2011), it was shown that the phase information in the N100 is a more robust indicator of habituation than the amplitude. These phase changes are thought to reflect lower attentional-binding to the stimulus such that there is an increased jitter in the phase with habituation (Low and Strauss, 2011; Mortezapouraghdam et al., 2014). Figure 1 shows the changes in the phase information of attention-correlate N100 wave for a soft (easy to habituate) and aversive (difficult to habituate) stimulus.

In Strauss et al. (2013), a 2D denoising algorithm was applied to the amplitude of collectively segmented ERPs to extract the morphological signature of the habituation effect in the data. Measures such as the time-scale coherence between ERP trials were used in Mariam et al. (2009) to evaluate the effect of habituation. Most of the preceding studies use the amplitude or amplitude-phase domain to understand the effect of habituation, however in Low and Strauss (2011) and Strauss et al. (2008), it was reported that fewer trials were required to distinguish between the habituation and non-habituation processes using only the phase information of ERPs across the responses.

In a recent study in Mortezapouraghdam et al. (2014), the effect of long-term habituation was solely based on the study of instantaneous phase information of ERPs for two different stimuli levels. The two stimuli consisted of pure-tones of 50 and 100 dB SPL (The 50 dB SPL is considered as a soft stimulus, as it is easy to habituate to the sound and 100 dB SPL is considered as aversive stimulus with no habituation effect). The effect of habituation was evaluated by fitting a parametric model to different data segments and tracking the data distribution over the time. Based on average rate of data dispersion of segmented data, the study was able to objectively classify between habituation and non-habituation data from the recorded ERPs.

In this study, we introduce a Bayesian model which is able to infer the changes in the underlying probability distribution of circular data over time. Our method allows incorporating prior knowledge about the level of data dispersion and its behavior over time. The proposed approach is related to the Bayesian change-point algorithm which was presented in Paquet (2007) and Knill and Pouget (2004).

We compare our method to a moving-window-based maximum-likelihood estimation of the underlying distributions using generated data in Section 3.1. We furthermore use our method to assess the level of habituation on instantaneous phase information of ERPs obtained from 19 healthy subjects. The loudness levels used for the auditory stimuli in this data set range from a soft (and easy to habituate) 60 dB to a loud (and hard to habituate) 90 dB. Besides the methodology we use in this work, the range of the stimuli we analyze in the phase domain in this study (60, 70, 80, 90 dB SPL) is one of the major differences with our previous studies (Strauss et al., 2008; Mariam et al., 2009; Mortezapouraghdam et al., 2014). The classification between different ERPs for close-range stimuli is more difficult as the elicited ERPs are more similar and hence more sophisticated methods are required to capture the subtle differences. We evaluate the performance of our method as a discriminator between different loudness levels in Section 4.

## 2. Materials and methods

In this section we describe: (1) A brief description of data acquisition details and pre-processing of ERP images using non-local-means algorithm (NLM), (2) Extraction of instantaneous phase of ERP images using wavelet transformation.

### 2.1. Human data acquisition

Twenty participants (16 female and 4 male; mean age: 23 years and 3 months with a standard deviation of 4 years and 1 month) attended the experiment. One subject's data was lost due to data corruption and the following focuses on the remaining 19. All participants had normal hearing as assessed by an audiogram test before and after the experiment. All subjects provided informed consent and the study was conducted in accordance with the declaration of Helsinki. The data was recorded during a training session for students focusing on intensity relations on event-related potentials. The aim of the training session was to see amplitude and latency changes of the N1 wave related to different intensities of stimuli (See chapter 12 in Hall, 2007).

Before the electrophysiological experiment, the subjective loudness measurement was measured. Auditory stimuli were presented in 10 different auditory levels from 10 to 90 dB SPL for a few trials and the subjects were asked to scale the loudness of the stimuli based on 10 different scaling levels (not heard, threshold, very soft, soft, comfortable but soft, comfortable loud, comfortable but loud, loud upper level, and too loud). To obtain reliable feedback about the loudness of the stimuli, we used healthy subjects with no previous hearing abnormalities.

For the electrophysiological experiment, subjects lay on an examination bed and were instructed to relax with closed eyes. The EEG signals were recorded using surface electrodes (Ag/AgCl) which were placed at the right and left mastoid (active), the vertex (reference) and the upper forehead (ground). The signal acquired from each mastoid was referred to vertex (Cz) and processed separately. The electrode impedance was kept below 5 *k*Ω and the recording EEG was sampled at 512Hz.

The experiment consisted of listening to a series of pure tone beeps presented in their right ear via headphones (Sennheiser HDA 200) at four different volumes of 60, 70, 80, and 90 dB. Each beep was at a frequency of 1 kHz and lasted 40 ms. There was a 1 s ISI between stimuli and at least 500 tones were presented per stimulation level. Participants were instructed not to pay attention to the stimulus and attempt to ignore the sound during the experiment. To be sure they did not fall asleep, they were constantly monitored and checked to be awake.

### 2.2. Data pre-processing

We preprocessed our data in three steps outlined in detail below. First we segmented the data into ERPs centered on the presentation of the tones. Next we denoised the data using a non-local means algorithm. Finally, we extracted phase information from the data using a wavelet transform.

### 2.3. Data segmentation

To remove unwanted frequencies, we applied a bandpass FIR filter of order 1000 and cut-off frequencies of 1 and 30 Hz to the raw EEG. The high filter order was applied to minimize the phase distortion effects. We then segmented the data to focus on the first 800 ms (or at 512 Hz, *M* = 410 samples) post stimulus for each trial. Trials that contained amplitudes larger than 50μ*V* were assumed to contain artifacts and were removed. This process resulted in at least *N* = 439 artifact free trials $e_{k}^{T}\in {\mathbb{R}}^{\text{M}},k=1,\cdots ,N$ for each subject.

**Figure 2:**
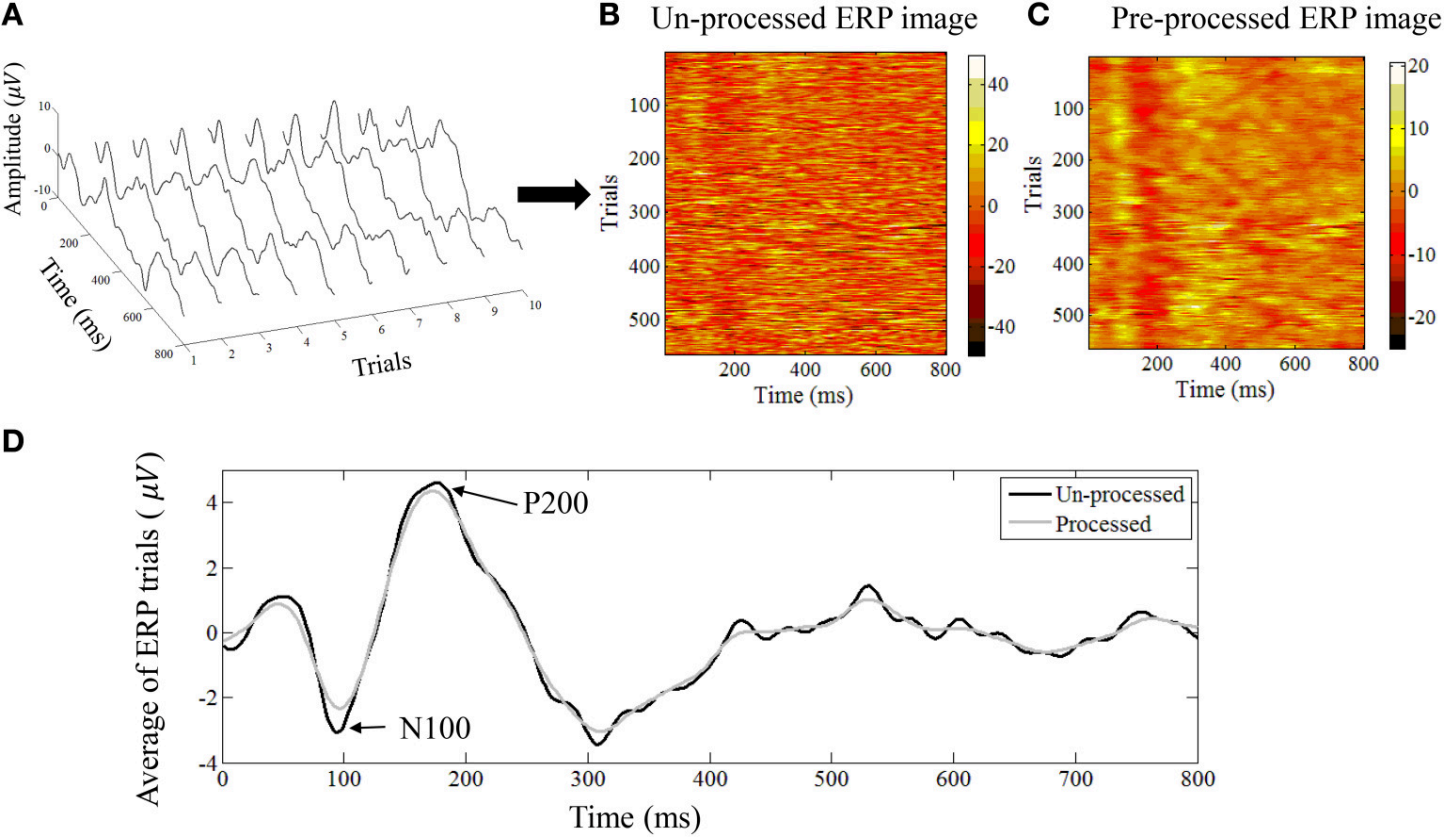
**(A)** An example of single trial auditory ERPs. Every trial corresponds to the post-stimulus response. **(B)** All trials can be represented as an ERP image where every row corresponds to one trial. **(C)** The denoised version of the ERP image after applying the NLM algorithm. **(D)** The average ERP of all trials before and after applying the NLM. The N100 negative evoked potential peaks between 80 and 100 ms after the onset of stimulus in adults. Its amplitude is strongly correlated with the time of stimulus onset, stimulus loudness, and inter-stimulus loudness with other sounds. The N100 is followed by the P200 positive evoked potential (usually referred to as the N1-P2 complex).

We represent the *N* trials by *M* samples per trial ERP image as

$$E\;:\text{​}={\left(e_{1},\ldots ,e_{N}\right)}^{\text{T}}$$

where $e_{k}\in {\mathbb{R}}^{\text{M}}$ are the post-stimulus trials that appear as the rows in the matrix. In Figures 2A,B we show an example of 60 dB (SPL) single-trial ERPs along with the ERP image representation.

### 2.4. Data denoising

To denoise the data we applied a non-local means (NLM) algorithm to the two dimensional ERP images. The NLM algorithm was first developed by Efros et al. for denoising texture synthesis (Efros and Leung, 1999). In particular, this algorithm takes advantage of the extensive self-similarity and repetition in some textures to smooth the data using image neighborhoods—or patches—that represent local structure.

Since our ERP images also contain a high level of self-similarity—because of the time-locked stimulation (Strauss et al., 2013)—we have previously shown that the NLM algorithm is a good way to denoise our data while maintaining the regularities between multiple trials (Strauss et al., 2013). In particular, by adjustment of the parameters, we found that this denoising algorithm allowed us to extract the morphological changes in the relative amplitude or latency changes of the single trials and yield physiologically meaningful information, such as amplitude modulations of the N100 wave.

Here we used the exact same denoising parameters as in Strauss et al. (2013) to denoise the ERP image *E* ∈ ℝ^N × M^ into the denoised ERP image *Q* ∈ ℝ^N × M^, whose rows ${\text{q}}_{k}\in {\mathbb{R}}^{\text{M}}$ represent the denoised ERP from trial *k*. In Figures 2B,C we show an example of ERP image before and after NLM denoising. The averaged ERP before and after denosing is shown in Figure 2D.

### 2.5. Phase extraction

To compute the instantaneous phase information of the denoised trials ${\text{q}}_{k}\in {\mathbb{R}}^{\text{M}},k=1,\ldots \text{N}$ we employ continuous wavelet transform as explained in Low and Strauss (2011) (See Bruns, 2004 for further studies about different mechanism on extracting the phase information and the similarity between them). For every ${\text{q}}_{k}\in {\mathbb{R}}^{M},k=1,\ldots \text{N}$ we compute the complex wavelet coefficients as

$$\omega_{k,b}\;:\text{​}={\langle q_{k},\psi_{b}^{a}\rangle }_{L^{2}},\;b=1,\ldots ,M,$$

where ψ ∈ *L*^2^ (ℝ) is the wavelet function with *a, b* ∈ ℝ, *a* ≠ 0 representing the *scale* and *translation* parameters, respectively.

We choose the wavelet function to be the sixth derivative of the complex Gaussian where $\psi_{b}^{a}$ is the sampled vector of

$$\psi_{b}^{a}(x)\;:\text{​}=|a{|}^{-1/2}\psi ((x-b)/a)).$$

It has been previously illustrated that a fixed scale *a* = 40 can be used reliably (significance of *p* < 0.05) to show physiological meaningful correlates of N100 modulations of auditory ERPs for analyzing the attention correlate (Strauss et al., 2008; Low and Strauss, 2011). By applying the wavelet transformation for a fixed scale *a* and discretized translations *b*_*m*_(*m* = 1, 2, …, M), we introduce the mapping G_*a, b*_ : **Q** ↦ **P** (**P** ∈ ℝ^N×M^) with the complex entries of ω_*k, b*_. From the complex entries

$$\omega_{k,b}=Re(\omega_{k,b})+iIm(\omega_{k,b})=|\omega_{k,b}|\exp(ip_{k,b})$$

with absolute value $|\omega_{k,b}|\;:=\sqrt{Re{\left(\omega_{k,b}\right)}^{2}+Im{\left(\omega_{k,b}\right)}^{2}}$ we obtain the phase *p*_*k, b*_ by

(1) $$\begin{array}{c} \begin{array}{l} p_{k,b}\;:\text{ ​}=\text{atan2}(Im(\omega_{k,b}),Re(\omega_{k,b}))\\ \;=\{\begin{array}{cc} \arccos\frac{Re(\omega_{k,b})}{\left|\omega_{k,b}\right|} & \text{if }Im(\omega_{k,b})>0,\\ -\arccos\frac{Re(\omega_{k,b})}{\left|\omega_{k,b}\right|} & \text{if }Im(\omega_{k,b})\leq 0. \end{array} \end{array} \end{array}$$

Here atan2 denotes the “quadrant–specific” inverse of the tangent function. The definition enforces the 2π-periodic angles *p*_*k, b*_ to be in ∈ [−π, π). Hence by computing the phase $p_{k}^{T}=(p_{k,1}$ of every trial **q**_*k*_), we construct the instantaneous phase matrix

$$P={\left(p_{1},p_{2},\ldots ,p_{N}\right)}^{\text{T}}$$

of the ERP image **Q**. As described before in the Introduction, Figure 1 shows an example of the instantaneous phase matrix. We study the effect of habituation over phase trials at seven distinct times denoted as **Θ**_(*t*)_ = {θ_1_, …, θ_*N*_}, θ_*i*_ ∈ [−π, π), θ_*i*_ ∈ *p*_*i, t*_∀_*i* = 1…*N*_ where the time span is from *t* = 97*ms* to *t* = 127*ms* after the onset of stimulus.

### 2.6. Data modeling description

In this section: (1) We briefly describe the method of estimating the concentration parameter of a von Mises distribution at different segments of phase data. The explained method is based on previous work. (2) We introduce the forward-backward Bayesian model as an appropriate method for detecting the exact times of change. (3) We fully describe the details of the model and criteria for determining the model parameters.

### 2.7. Maximum likelihood estimate of concentration parameter

In a previous study (Mortezapouraghdam et al., 2014), we looked at the auditory selective attention correlate dynamics for soft (50 dB SPL) and aversive (100 dB SPL) sound stimuli by measuring the distribution of instantaneous phase data over trials at a fixed time of interest *t* (as shown in Figure 1). In particular, we found that soft stimuli were associated with a broader distribution of phases than aversive stimuli, when the distribution of phases was more concentrated. The results indicate the low binding of the attention to the soft stimulus and high binding of attention to the aversive stimulus.

**Figure 3:**
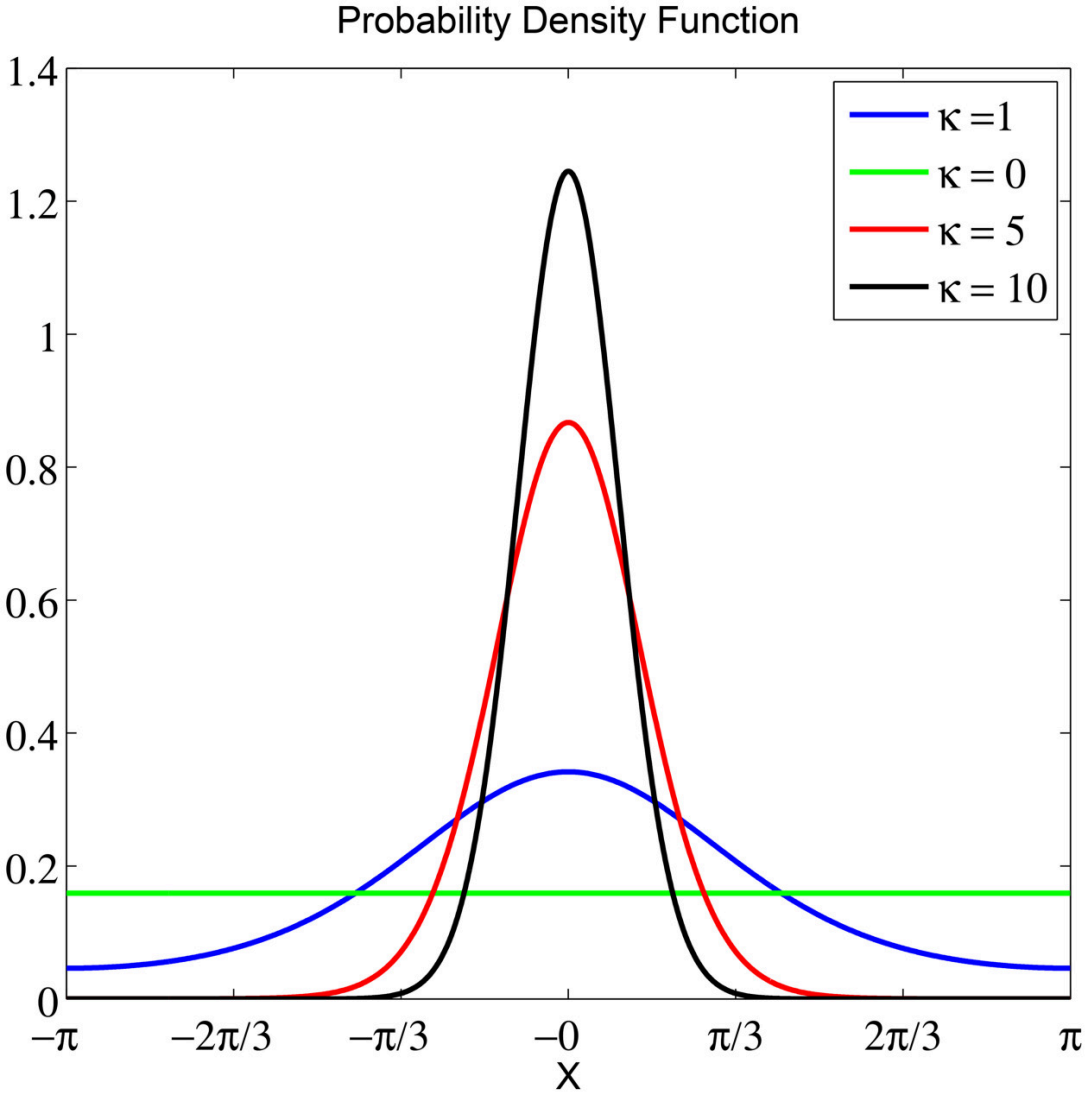
**The probability density function of a von Mises distribution with μ = 0 and different concentration values κ**. For the smallest κ = 0, the distribution is uniform. As κ increases, the distribution becomes very concentrated around the angle μ.

To obtain an objective measure for classifying between the two stimuli based on instantaneous phase information, we split the data into sliding windows of *G* trials with overlap of *g* and performed a fit with a von Mises distribution for each window. The von Mises distribution is known as one of the main popular parametric models for the analysis of *directional data* (Fisher, 1995). It is analog to a normal distribution of real-valued data. The von Mises distribution is defined as $f\left(\theta ;\mu ,\kappa \right)={\left(2\pi I_{0}\left(\kappa \right)\right)}^{-1}\exp\left(\kappa \cos\left(\theta -\mu \right)\right)$ which consists of a mean μ ∈ [−π, π) that determines its location, a concentration parameter κ ∈ ℝ^+^ that determines its width and *I*_0_(κ) is a modified Bessel function of first kind and order r. For more information on the maximum likelihood estimate of the parameters of a von Mises distribution and its properties see Jammalamadaka and SenGupta (2001) and Fisher (1995). As κ increases, the distribution has a high kurtosis, following a high concentration of data samples. See Figure 3 for illustrations of von Mises distributions with different value of concentration (κ) and a zero mean (μ = 0). The smallest κ = 0 resembles a uniform distribution.

There are a number of limitations with the assessment of phase information using the latter approach. A small window size makes the results susceptible to noise in the input, and can lead to over-fitting. A large window size on the other hand comes at a loss of time precision, making it impossible to detect the exact time at which a change in the underlying data distribution occurs. The method in general lacks the ability to detect the gradual change in the habituation process with a high temporal precision. In addition it is not easily possible to incorporate prior knowledge about the long-term habituation process of attentional circuits into the model. Incorporating such information can be useful for making the results more robust against noise, and to therefore improve the performance of certain classification tasks (see Section 4 for an example).

**Figure 4:**
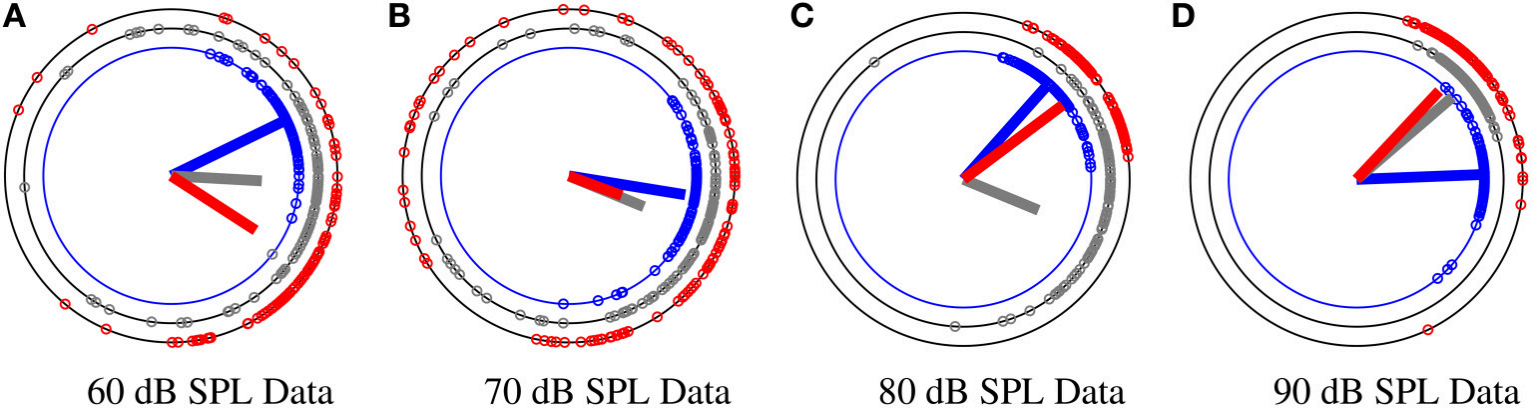
**An example of phase distribution over trials at a specific time ***t, t*** = 97 ms for a subject at four different stimuli levels of 60, 70, 80, and 90 dB SPL**. The phase data Θ_*t*_ has been divided into three different segments corresponding to the beginning (BI), middle (MI), and end (EN) of the experiment. Segment size is *G* = 150. Blue, gray, and red data circles correspond to the BI, MI, and EN of the experiment, respectively. The lines correspond to the resultant mean vector divided by the number of segment samples. As phase data spread more around the circle, the length of the vector mean decreases. **(A)** The distribution of phase samples corresponding to 60 dB SPL at different segments BI, MI, EN. **(B)** The distribution of phase samples corresponding to 70 dB SPL at BI, MI, EN. **(C)** The distribution of phase samples corresponding to 80 dB SPL at BI, MI, EN. **(D)** The distribution of data samples corresponding to 90 dB SPL at BI, MI, EN.

To obtain a more precise and flexible approach for detection of habituation effect, we propose a Bayesian modeling approach for directional data which is able to capture the changes in the data with a higher time resolution. Prior knowledge about the underlying data can be easily incorporated into our model and improve the reliability of the fitted posterior distribution under noise. In Section 2.8 we present the details of our model and validate the proposed model on artificial directional data in Section 3. At last, we use the method as an objective measure on phase information of the N100 attention-correlate wave at four different stimuli levels of 60, 70, 80, 90 dB SPL for detecting the level of habituation. We classify between the four different stimuli in Section 3. In Figure 4 we show an example of the phase modulation for different stimulus at different segments *G* = 150 and *g* = 0. It is observed that the mean direction for low stimuli levels (60 and 70 dB SPL) changes more in comparison to the higher stimulus levels (80 and 90 dB SPL).

### 2.8. Bayesian model

Here we derive a recursive Bayesian estimation for modeling the changes in the hidden states that explain and generate the time series data. This model computes the likelihood of all the possible values of the random variable(s) at every stage. This allows the system to integrate information efficiently over time and space, and to propagate information from one stage to another without having to draw concrete conclusions at early stages (Knill and Pouget, 2004).

In our Bayesian model, the sequence of phase observations is defined over phase trials at a specific time *t* (as illustrated in Figure 1), more precisely **Θ** = {θ_1_, θ_2_, …, θ_*N*_}, θ_*i*_ ∈ [−π, π). The data is modeled by assuming that at each time step, θ_*i*_ was generated from a certain but unknown state variable S_*t*_. Here we define S_*t*_ = (μ_*t*_, κ_*t*_) by the parameters of a von Mises distribution, where μ_*t*_ and κ_*t*_ are the mean and concentration parameter, respectively. For simplicity we represent this state on a grid of discrete values R_μ_ = {*u*_1_, ⋯ , *u*_*m*_}, *u*_*i*_ ∈ [−π, π) and R_κ_ = {*k*_0_, ⋯ , *k*_*m*_}, *k*_*i*_ ∈ [0, ℓ] where ℓ is the upper-bound of the concentration discretization.

We assume that we have prior information on how the states should evolve given the past states *p*(S_*t*+1_|S_1_, ⋯ , S_*t*_), but we cannot observe them directly. The available information is the measurements that are dependent on the state, but noisy: *p*(θ_*t*_, ⋯|S_*t*_).

The assumptions made in the Bayesian network are as follows:

1. The observation at time *t* was generated by some process whose state S_*t*_ is hidden from the observer.
2. The states satisfy the first order Markov property. That is given the value at S_*t*−1_, the current state S_*t*_ is independent of all states earlier than *t*−1, that is *p*(S_*t*_|S_1_, ⋯ , S_*t*−1_) = *p*(S_*t*_|S_*t*−1_).
3. The observations are conditionally independent given the current state S_*t*_. That is *p*(θ_*t*_|θ_1:*t*−1_, S_*t*_) = *p*(θ_*t*_|S_*t*_) (the Markov property of data with respect to the states).

Our goal is to infer the distribution over hidden states (μ_*t*_, κ_*t*_) based on the phase data from all *N* trials, θ_1:*N*_. Given the conditional independence in our model, this is straightforward to write down as:

(2) $$\begin{array}{l} p(\mu_{t},\kappa_{t}|\theta_{1:N})\propto p(\theta_{t+1:N}|\mu_{t},\kappa_{t})p(\mu_{t},\kappa_{t}|\theta_{1:t}), \end{array}$$

where *p*(μ_*t*_, κ_*t*_|θ_1:*t*_) denotes the distribution over states at time *t* given information in the past and *p*(μ_*t*_, κ_*t*_|θ_*t*+1:*N*_) is the distribution over the states given information in the future. These distributions are easy to compute using recursive algorithms that sweep forwards (for *p*(μ_*t*_, κ_*t*_|θ_1:*t*_)) and backwards (for *p*(μ_*t*_, κ_*t*_|θ_*t*+1:*N*_)) through the data. We describe these sweeps in the following sections.

#### 2.8.1. Forward pass

**Figure 5:**
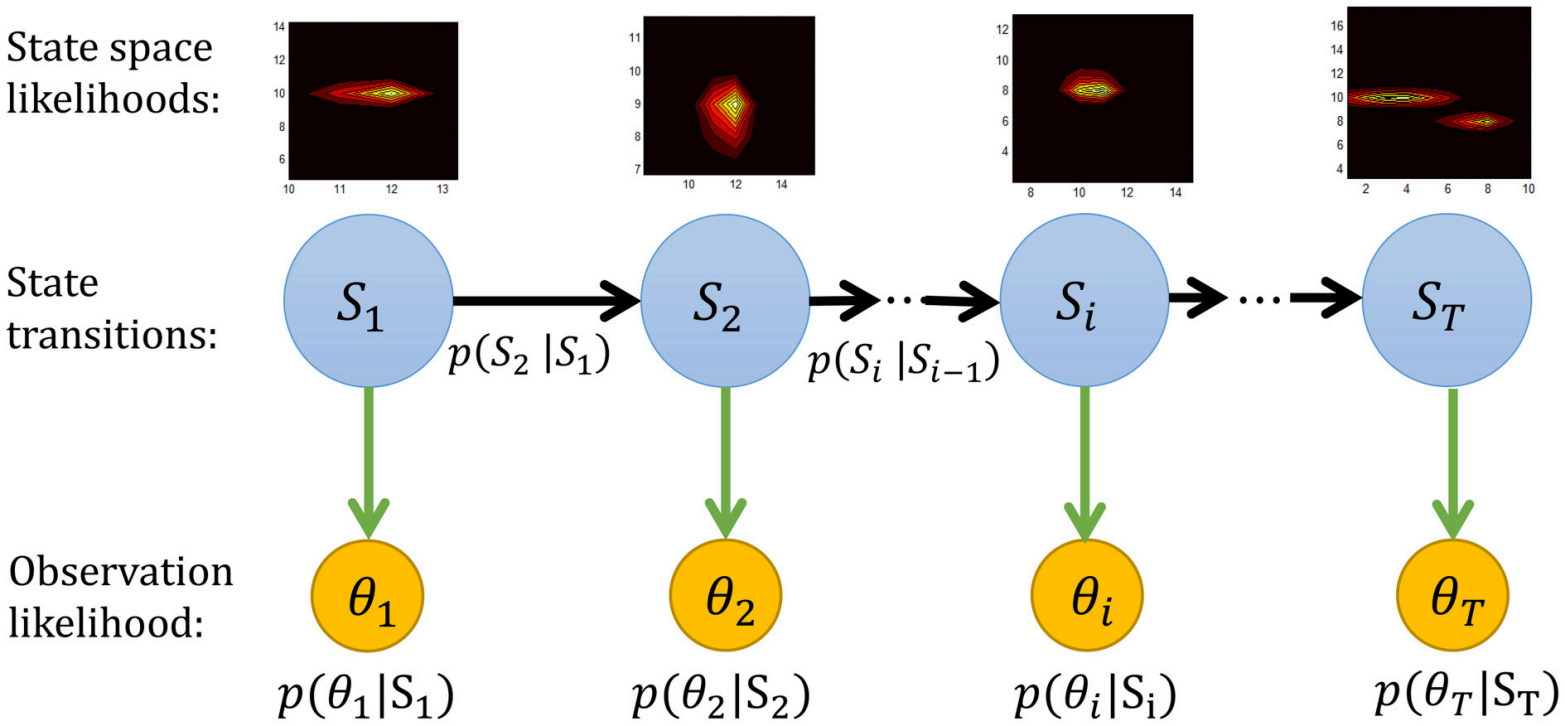
**The graphical representation of the forward Bayesian model**. The state space likelihood in our study are the mean μ and concentration κ parameters of a von Mises distribution. More precisely *S*_*i*_ = (μ_*i*_, κ_*i*_).

The forward sweep computes *p*(μ_*t*_, κ_*t*_|θ_1:*t*_), the distribution over the state at time *t*, given data in the past. Given the dependencies outlined in Figure 5, this can be computed recursively using Bayes rule as

(3) $$\begin{array}{ccc} p(\mu_{t},\kappa_{t}|\theta_{1:t}) & \propto & p(\theta_{t}|\mu_{t},\kappa_{t})p(\mu_{t},\kappa_{t}|\theta_{1:t-1}) \end{array}$$

(4) $$\begin{array}{l} =p(\theta_{t}|\mu_{t},\kappa_{t})\sum_{\mu_{t-1},\kappa_{t-1}}p(\mu_{t},\kappa_{t}|\mu_{t-1},\kappa_{t-1})\\ p(\mu_{t-1},\kappa_{t-1}|\theta_{1:t-1}) \end{array}$$

where *p*(θ_*t*_|μ_*t*_, κ_*t*_) is the likelihood of the data given the parameters and *p*(μ_*t*_, κ_*t*_|μ_*t*−1_, κ_*t*−1_) defines our prior on the dynamics of μ_*t*_ and κ_*t*_. This term will be discussed in detail in Section 2.8.4 below.

#### 2.8.2. Backward pass

The backward pass computes the probability of the future data given the state at time *t*, *p*(θ_*t*+1:*N*_|μ_*t*_, κ_*t*_). As with the forward pass, this can be computed recursively using Bayes rule

(5) $$\begin{array}{l} p(\theta_{t+1:N}|\mu_{t},\kappa_{t})=\sum_{\mu_{t+1},\kappa_{t+1}}p(\mu_{t+1},\kappa_{t+1}|\mu_{t},\kappa_{t})\\ \;p(\theta_{t+1:N}|\mu_{t+1},\kappa_{t+1}) \end{array}$$

(6) $$\begin{array}{l} =\sum_{\mu_{t+1},\kappa_{t+1}}p(\mu_{t+1},\kappa_{t+1}|\mu_{t},\kappa_{t})\\ p(\theta_{t+1}|\mu_{t+1},\kappa_{t+1})p(\theta_{t+2:N}|\mu_{t+1},\kappa_{t+1}) \end{array}$$

where *p*(θ_*t*+1_|μ_*t*+1_, κ_*t*+1_) is the same likelihood and *p*(μ_*t*+1_, κ_*t*+1_|μ_*t*_, κ_*t*_) the same prior as used in the forwards pass.

#### 2.8.3. Forward-backward model and the initial conditions

**Figure 6:**
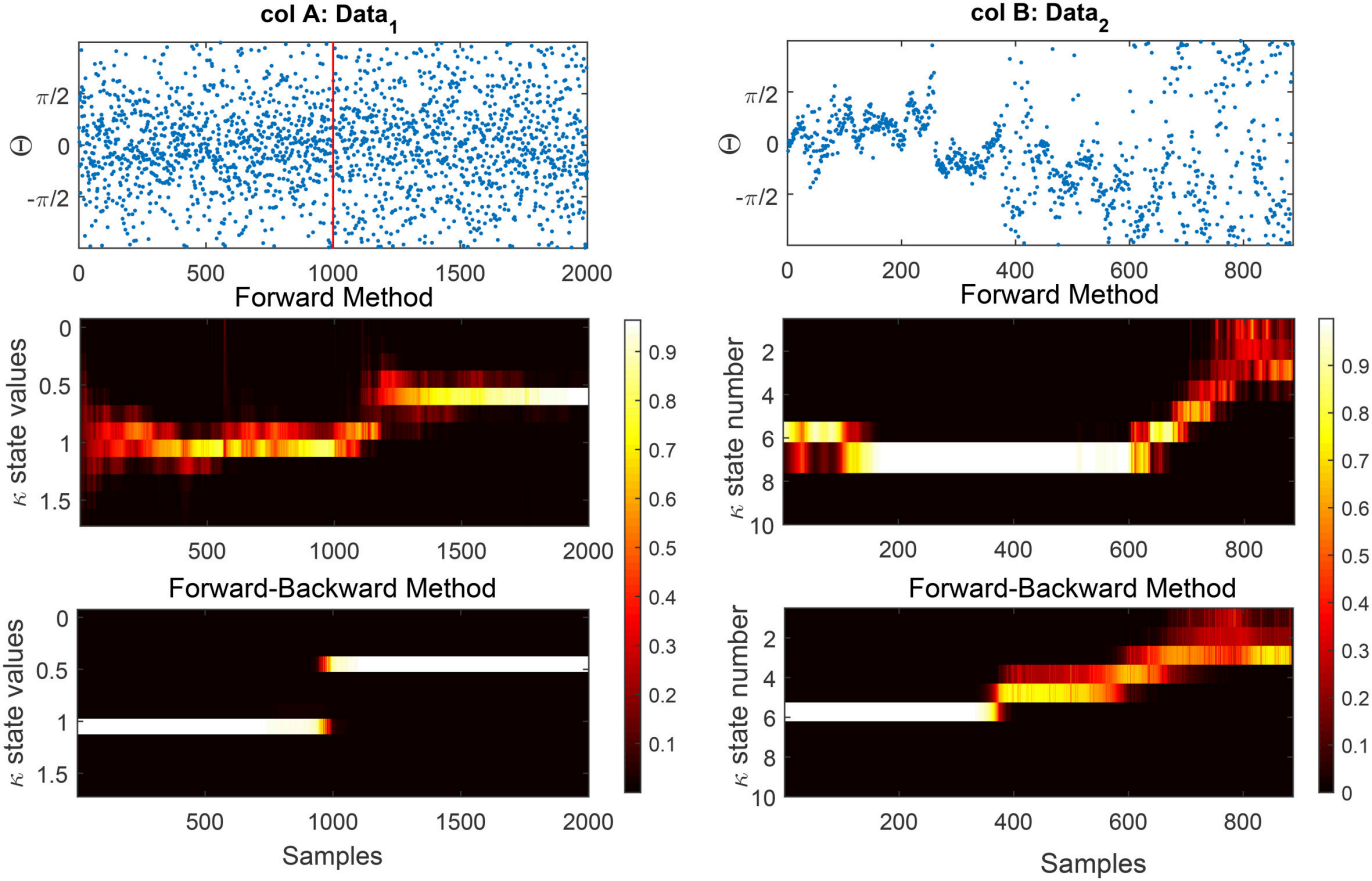
**We show the effect of the forward and forward-Backward approach for two different data examples**. **Column A**: shows an artificial data set generated using a rejection sampling approach with an exact time of change at *t* = 1000. The estimated concentration has been shown for the forward and forward-backward approach, respectively. **Column B**: shows the estimated concentration for the forward method (1B) and (2B) of phase information of habituation data. The effect of the initialization on the results of the forward method is clearly visible. In terms of change detection in the phase modulations, the forward-backward approach provides more accurate timing information than the forward approach which occurs near *t* = 400.

One problem of the forward method is that its results are heavily influenced by the initialization at *t* = 1. Depending on how the state likelihoods for the first observation are initialized, state likelihoods at subsequent times can be far from the globally optimal explanation of the data (see Figure 6 for the effect of non-adequate initialization in data). A similar effect is observed if there is a sudden change in the underlying states at a later time in the data or if the initial data points are outliers (noise). Because the forward method does not *look ahead* of the current time, it adapts to changes in the data only with a delay. We add a backward pass to compensate for these effects, and to remove any influence of the *direction of time* on the results.

We also mitigate the initialization problem by using the result of the forward pass at *t*_*N*_ to initialize the backward pass, and vice-verse. Unless the data distribution drastically changes very close to the end of the data *t*_*N*_, the forward method usually has sufficiently converged at that point, thus providing a very stable initialization for the backward pass. After the backward pass is done, we run the forward pass for a second time, using the result at *t*_1_ from the backward pass for its initialization. Only the results of the second forward pass together with the results of the backward pass are finally used. The results of the first, randomly initialized forward pass are discarded in the final result, thereby removing most influence of the original random initialization.

#### 2.8.4. State transition model, *p*(μ_*t*_, κ_*t*_|μ_*t*−1_, κ_*t*−1_)

For the prior distribution over the state transitions *p*(μ_*t*_, κ_*t*_|μ_*t*−1_, κ_*t*−1_), we first assume that the transition distributions for the mean, μ, and concentration, κ, are independent; i.e.,

$$p\left(\mu_{t},\kappa_{t}|\mu_{t-1},\kappa_{t-1}\right)\propto p\left(\mu_{t}|\mu_{t-1}\right)p\left(\kappa_{t}|\kappa_{t-1}\right).$$

For transition distributions of μ and κ we choose von Mises and Gaussian distributions, respectively.

More specifically, for the circular parameter, μ_*t*_ ∈ [−π, π) we assume a von Mises distribution with the mean of μ_*t*−1_ and the concentration of *K* ∈ ℝ^+^; i.e.,

$$p\left(\mu_{k}|\mu_{k-1},K\right)=\frac{\exp\left(K\cos\left(\mu_{t}-\mu_{t-1}\right)\right)}{2\pi I_{0}\left(K\right)}.$$

For the real valued concentration random variable, we assume a Gaussian transition distribution,

$$p\left(\kappa_{t}=\kappa_{t}|\kappa_{t-1}=\kappa_{t-1}\right)=\frac{1}{\sqrt{2\pi }\sigma }\exp\left(\frac{-{\left(\kappa_{t}-\kappa_{t-1}\right)}^{2}}{2\sigma^{2}}\right)$$

with variance σ^2^ > 0.

As the states are discrete random variables, for computing the *p*(μ_*t*_|μ_*t*−1_) and *p*(κ_*t*_|κ_*t*−1_), we evaluate the von Mises distribution and Gaussian distribution for all possible values of μ_*t*_ and κ_*t*_ and then normalize it.

The selection criteria for the prior parameters *K* and σ^2^ for measured data is described in Section 2.8.5.

#### 2.8.5. Optimization of prior parameters

In this section we report on the setting of the model prior parameters *K* and σ^2^ and other parameters which were used for analyzing the phase information of ERPs corresponding to 19 subjects at different stimulus levels of 60, 70, 80, and 90 dB SPL. The phase information over trials was analyzed at seven distinct times of **Θ**_*t*_ (*t* = 97 to *t* = 197*ms* with step sizes of three, or more specifically for all trials at a specific sample of *M* = 44 to *M* = 62) for the phase matrix P ∈ ℝ^*N*×*M*^. The number of discrete states for R_μ_ and R_κ_ is *m* = 20 with an upper bound of ℓ = 63 for the concentration parameter. We discretize the concentration parameter κ using a logarithmic scale.

The state transition model described in Section 2.8.4 has two free parameters, *K* and σ^2^ which determine the speed with which μ and κ change over time, respectively. If we fit the parameters such that the likelihood of the observations *p*(Θ_1:*T*_) is maximized we overfit the model to a specific dataset. The main problem with this approach is that it does not help us to discriminate between different groups of stimuli as reliably as possible. In addition, no prior information regarding the decaying behavior of the habituation process is used. We propose to set the free parameters *K* and σ^2^ such that the resulting fits are robust under noise, and allow us to discriminate between different stimulus levels, or more precisely to reliably predict the stimulus level that the subject has been exposed to. The optimization criteria for the prior parameters is defined such that the ratio of the variance between different groups of stimulus levels to the variance of within groups of our novelty measure is maximized as defined in Equation (7). Here the novelty measure is defined as the “normalized” expectation value $E^{\prime }\left(\kappa_{t}\right)$ which informs about level of change in the κ parameter rather than its absolute value.

(7) $$\arg\underset{K,\sigma^{2}}{\max}\left(\rho =\frac{\text{between group variance of }E\prime (\kappa_{t})}{\text{average within group variance of }E\prime (\kappa_{t})}\right)$$

The procedure for computing the normalized expected value $E^{\prime }\left(\kappa_{t}\right)$ of concentration κ is as follows:

For a given observation **Θ** we first obtain $E\left(\kappa_{t}\right)=\sum_{\kappa_{j}\in \kappa }\left\{\kappa_{j}p\left(\kappa_{t}=\kappa_{j}|\Theta ,\sigma^{2},K\right)\right\}\forall t=1\cdots N$ by performing the forward-backward passes. Since our primary interest lies in the change of κ over trials, and not in its absolute value, we apply a normalization as follows: Given the sequence of *E*(κ_*t*_) over time *t* for a given subject and stimulus, we compute the average of the last 50 samples. We then divide all values *E*(κ_*t*_) by the computed average: $E^{\prime }\left(\kappa_{t}\right)=\frac{E\left(\kappa_{t}\right)}{\overset{N}{\underset{i=N-50}{\sum }}E\left(\kappa_{j}\right)}$. Our choice to use the last 50 samples for the normalization is based on the assumption that any habituation-related change in the values of κ_*t*_ will have happened before that point.

For the optimization itself, we consider a finite set of possible parameters *K*∈*R*__*K*__*prior*__ = {*K*_1_, *K*_2_, …, *K*_*r*_} and $\sigma^{2}\;=\;\in R_{\sigma^{2}}=\left\{\sigma_{1}^{2},\sigma_{2}^{2},\ldots ,\sigma_{r}^{2}\right\}$. This is based on prior information about the habituation process in which the changes are defined to occur steady in time. Thereby they are set such that the corresponding probability distributions for the mean and concentration parameters are peaky (small width), indicating the transition in mean and concentration states cannot happen abruptly. Given the range of possible values for *K* and σ^2^ as *R*__*K*__*prior*__ = {*K*_1_, *K*_2_, …, *K*_*r*_} and $R_{\sigma^{2}}=\left\{\sigma_{1}^{2},\sigma_{2}^{2},\ldots ,\sigma_{r}^{2}\right\}$ respectively, we obtain *r*^2^ different configuration prior pairs $\left(K_{i},\sigma_{j}^{2}\right),i,j\in 1,2,\ldots ,r$. For each of these configurations, we first perform the forward-backward procedure and then compute the ρ criteria. Finally we select those parameters which lead to the highest ρ value. In Algorithm 1 in the appendix we illustrate the whole procedure for learning the prior parameters.

## 3. Results

In this section we describe: (1) Validating the model (as described in 2.8) on artificial circular data. (2) An example to compare the advantage of the forward approach in comparison to the forward-backward method. (3) Applying the model on phase information of the N100 wave of experimental human-measured ERPs at different stimuli. (4) The results of the model transition parameters *K* and σ^2^ for experimental data. (5) The individual and average results for all subjects at different stimuli levels. (6) Classification between different stimuli levels based on the criteria in Equation (7).

### 3.1. Model validation on artificial circular data

We first validate the method presented in Section 2.8 on artificial data generated from a von Mises distribution with different concentration parameters κ. The artificial data is generated using a rejection sampling method **Θ** = [**Θ**^(1)^, **Θ**^(2)^, …, **Θ**^(*J*)^], such that $\Theta^{\left(i\right)}\sim vonMises(\mu_{i},\kappa_{i})\forall i=1,\ldots ,J$ and with **Θ** of length L. As we are not interested in the mean changes of data, we keep the mean parameters constant for all data segments **Θ**^(*i*)^.

**Figure 7:**
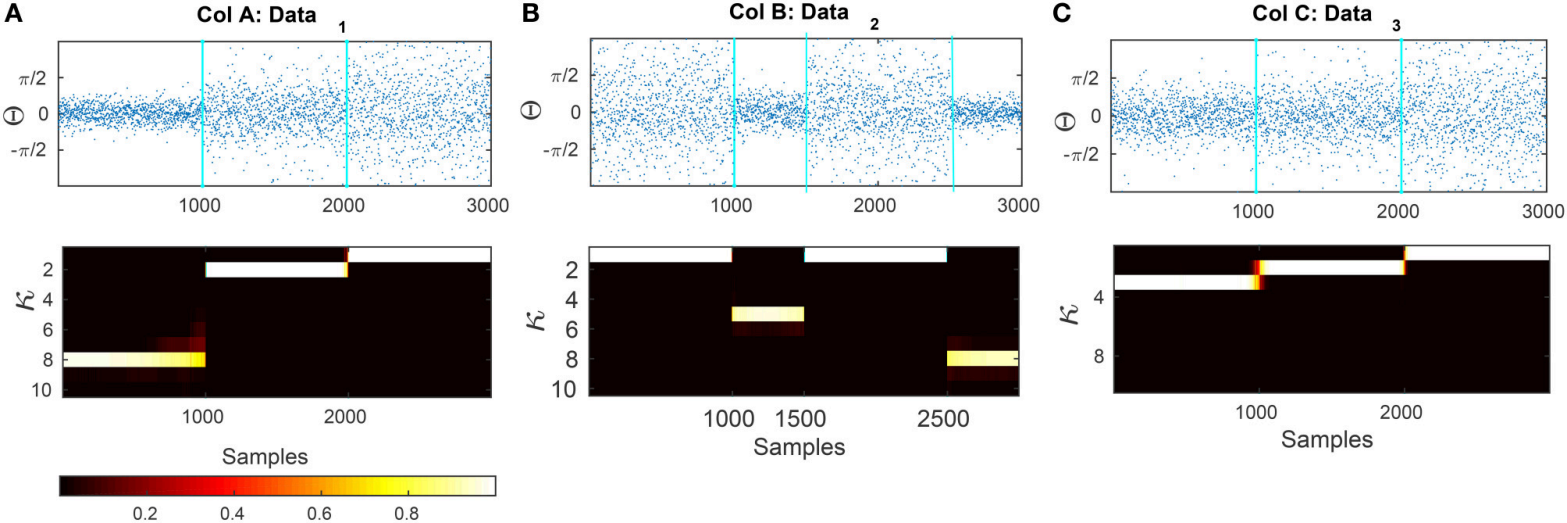
**Results of applying the forward-backward bayesian model on three different data sets**. **(A)** Artificial circular data that is generated from three different von-mises distributions with concentration parameters of κ_1_ = 8, κ_2_ = 2, and κ_3_ = 1. The transitions are at samples *t*_1_ = 1000 and *t*_2_ = 2000. **(B)** Circular data generated with dispersion values of κ_1_ = 1, κ_2_ = 5, κ_3_ = 1, κ_4_ = 8 with changes at samples *t*_1_ = 1000, *t*_2_ = 1500, and *t*_3_ = 2500. **(C)** Artificial data that is generated from κ_1_ = 3, κ_2_ = 2, and κ_3_ = 1. The transitions are at samples *t*_1_ = 1000 and *t*_2_ = 2000.

In Figure 7 we show two artificial data series that are composed of segments sampled from von Mises distributions with different κ parameters. The transition times between the distributions are at *t*_1_ = 1000 and *t*_2_ = 2000 for plot (a) and (b) and *t*_1_ = 1000, *t*_2_ = 1500 and *t*_3_ = 2500 for plot (c). After applying our Bayesian model, we compute the mean square error (MSE) of the estimated concentration and the actual concentration that was used for generating the data. For comparison, we also apply a maximum-likelihood approach to estimate the von Mises parameters using a moving window of different lengths (the ML estimation is described in Section 2.7). The MSE of our method for the data presented in Figures 7A–C are 0.0033, 0.014, and 0.033, respectively. We generated 50 artificial circular data sets with the same concentration parameters and transition points for each one of the examples Figures 7A–C and evaluated the average of MSE for the estimated concentration parameters. The MSE are 0.041 ± 0.04, 0.085 ± 0.064, and 0.010 ± 0.020, respectively.

**Figure 8:**
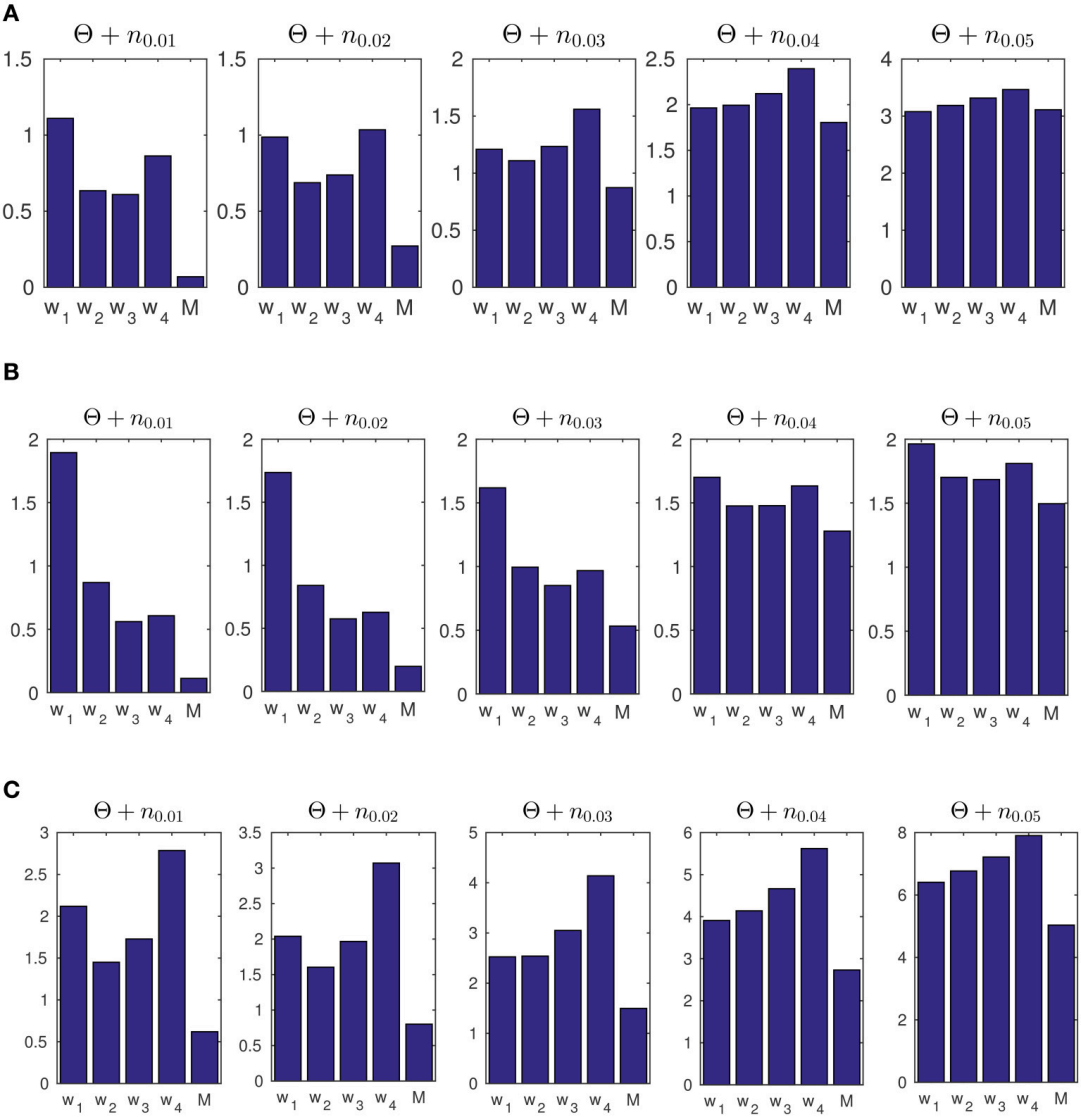
**(A)** The average of mean square error (MSE) of estimated concentration for 50 artificial data-sets. The generated data consists of concentration parameters of κ_1_ = 3, κ_2_ = 2, and κ_3_ = 1 with change points at times *t* = 1000 and *t* = 2000. The label M refers to the results obtained from our Bayesian forward-backward model. The labels *w*_1_ to *w*_4_ refer to different window sizes of 50, 100, 200, and 400 samples. **(B)** The MSE of 50 artificially generated data-sets with random transition times in the concentration parameter, and random κ values. **(C)** The MSE for 50 artificially generated data-sets with transition times at *t* = 1000 and *t* = 2000 with the concentrations of κ_1_ = 9, κ_2_ = 1, and κ_3_ = 9. We used the prior parameter σ^2^ = 0.6071 for $p\left(\kappa_{t}|\kappa_{t-1}\right)\sim N\left(\mu ,\sigma^{2}\right)$ for our model.

In addition, we tested the accuracy of our model and the ML approach on data set (c) in Figure 7 under additive noise, to assess the robustness of the methods. The noise was generated from a normal distribution with different variances $\Theta^{\left(i\right)}\sim vonMises(\mu_{i},\kappa_{i})\forall i=1,\ldots ,J$ for σ^2^ ∈ {0.01, 0.02, …, 0.05}. The resulting signal was wrapped to the range [0, 2π). For the windowing approach, we use four different sizes *G* = 50, 100, 200, 400 with 98% overlap as the moving window. The corresponding MSE results are shown in Figure 8A.

We used a prior of σ^2^ = 0.08 for Figure 7A. The small value of σ is due to the small range of concentration values in this example. Note that the transition are from κ → κ+1 in a single timestep. The transition probability of this event with the given σ is fairly low (about 0.0002), which avoids overfitting.

We furthermore tested the model on data with random change points and random concentration parameters. We generated 50 data-sets **Θ**, each with two random change points of different concentrations κ_1_ ∈ [6, 10], κ_2_ ∈ [3, 5] and κ_3_ ∈ [1, 2]. In each case we generated a series of *L* = 3000 sample points. We then computed the average MSE for both the windowing / ML approach and for our forward-backward approach. See Figure 8B. The same prior parameter as in part (a) was used for this part.

Note that for the windowing approach with window size *w*, an input of *L* samples only results in *L* − *w* parameter estimations. In order to make the MSE values comparable, we extended the input signal on both ends with $\frac{w}{2}$ additional samples from the first distribution, and the same number of additional samples from the final distribution.

In a third example in Figure 8C we generated data with a higher scale in its concentration, i.e., κ_1_ = 9, κ_2_ = 1 and κ_3_ = 9 for transition times at *t*_1_ = 1000 and *t*_2_ = 2000. We used the same prior parameter σ^2^ = 0.6071 for artificial data as was obtained for experimental data (In Section 3.3 we report on the results of the prior parameters for the experimental data). As shown, the model is able to track the data distribution at different noise levels. This example was used to show that given a similar range of concentration values of the data samples, the prior parameters from the experimental data can be used to track the distribution of artificial data-sets.

### 3.2. Comparison of forward and forward-backward estimation

As was described in Section 2.8.3, in Figure 6 we demonstrate the output of our model on two data sets using the forward and forward-backward approach. In column A we generate synthetic circular data of length 2000 with two different concentration parameters. The data up to time *t* = 1000 is generated from a von Mises with concentration 1 and it drops to 0.5 for the rest of the time-series. In column B we show the phase of N100 attention-correlate of auditory ERPs induced by low-decibel (soft) stimuli. This data illustrates the habituation effect as the phase distribution clearly gets broader around the 400th trial. This increase in the breadth of the phase distribution is well-captured by the concentration parameter in the model, which clearly shows an increase at trial 400.

### 3.3. Determining the model parameters for experimental data

**Figure 9:**
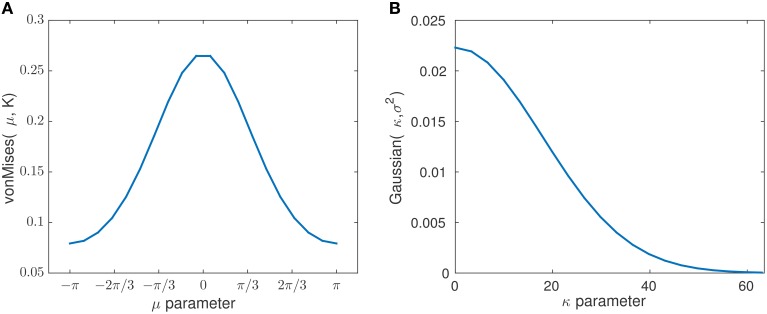
**The plots show the probability distribution for the mean and concentration states with chosen prior parameters ***K*** and σ^2^**. The plot **(A)** corresponds to the pdf (von Mises distribution) of the mean state transition model with the estimated concentration of *K* = 0.6071. The plot **(B)** corresponds to the pdf (Gaussian distribution) of the concentration state transition model with the estimated variance of σ^2^ = 320.

The prior parameters obtained for the transition models are set to *K* = 0.6071 and σ^2^ = 320. The criteria for optimizing over σ^2^ and *K* is described in Section 2.8.5 which is based on Equation (7). In Figure 9 we show the state transition probability distribution for the selected mean and concentration parameters *K* and σ^2^, respectively. These parameters play an important role on the speed of changes in the transition models and consequently, how abruptly the changes are determined. As an example, as the variance parameter σ^2^ in $N_{\mu ,\sigma^{2}}\left(x\right)$ increases and the concentration parameter *K* in *M*_μ, *K*_(*x*) decreases, the state transitions can occur more abruptly. This leads to a more abrupt and sudden detection of changes in the signal's phase distribution. In the extreme case of a uniform transition probability for the state transitions, at any time all subsequent states would have the same likelihood, independently of the current state. As a consequence, the μ_*t*_ state would simply track the signal itself, while leaving κ_*t*_ at a constant high level. In our case, the chosen parameters *K* and σ^2^ consider the prior information of habituation into the model.

### 3.4. Tracking data distribution over trials

**Figure 10:**
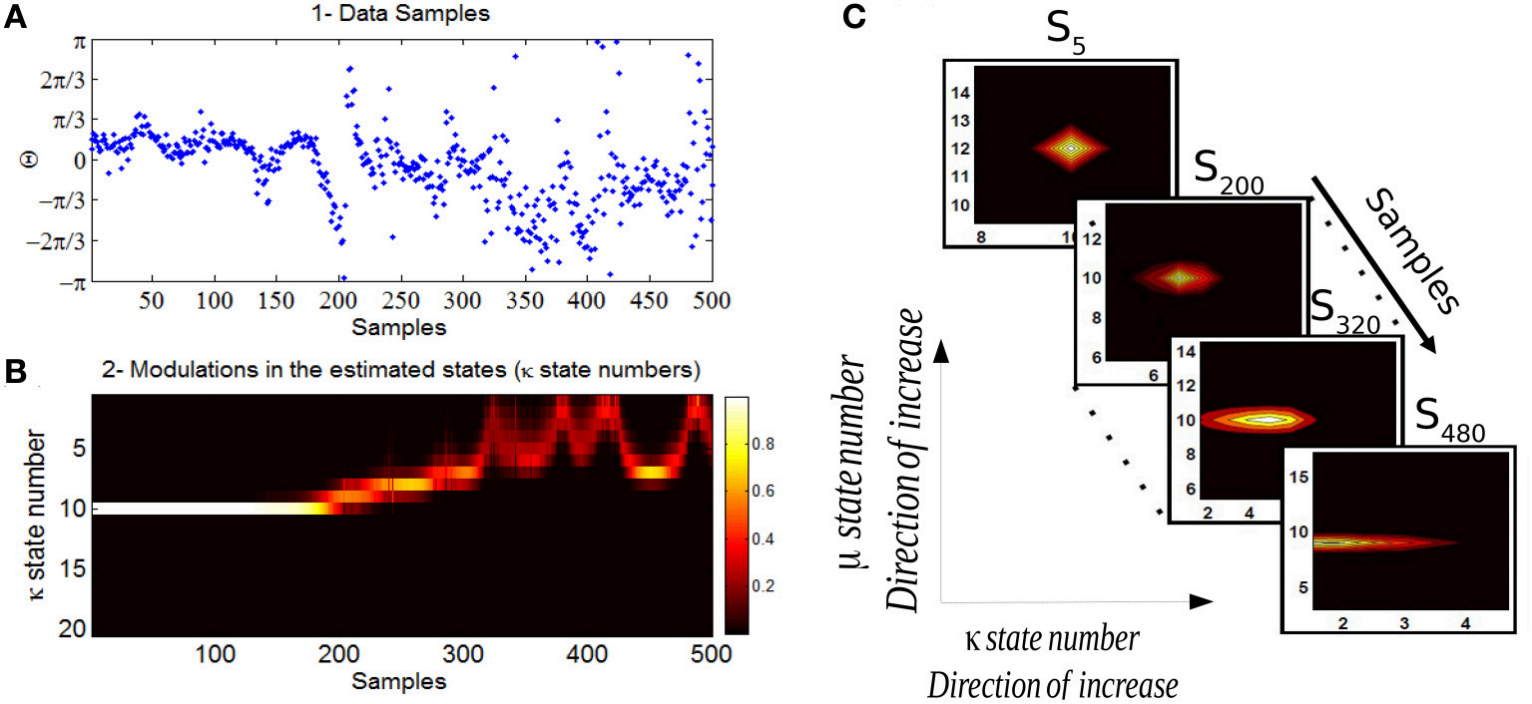
**(A)** The data corresponds to the phase information of auditory ERPs over trials for a case that habituation in long-term has been acquired. **(B)** The marginal likelihood of the concentration parameter over trials has been shown. **(C)** The individual likelihood values of the discrete state space at four different samples has been shown. The first state-space likelihood corresponds to the fifth sample (beginning of the experiment) up to the sample 480 which is the end of the experiment. The estimated concentration at the beginning of the experiment is high (reflecting a high binding of attention) and decreases significantly at the end of the experiment (lower binding of attention).

In Figure 10 we show the analysis of the Bayesian change point algorithm on a data in which a habituation is thought to be obtained. The data corresponds to 60 dB SPL which has in general a comfortable loudness perception. The likelihoods of the set of states in time (sample times) allow us to track the temporal changes in the mean and concentration parameters of the phase data. In this study we are particularly interested in the changes of the concentration parameter over trials which is interpreted as an indicator for the degree of attention allocated to the sensory stimulus. The corresponding marginal distribution of concentration parameter over all the samples has been shown in Figure 10B. Every state number (here it is between 1 and 20) corresponds to a different concentration values distributed logarithmically between [0,63]. In C the likelihood of the discrete state space at four sample times corresponds to samples from the beginning, middle, and end of the experiment. The level of the concentration intensity drops significantly at the end of the experiment in relation to its initial value at the beginning of the experiment.

**Figure 11:**
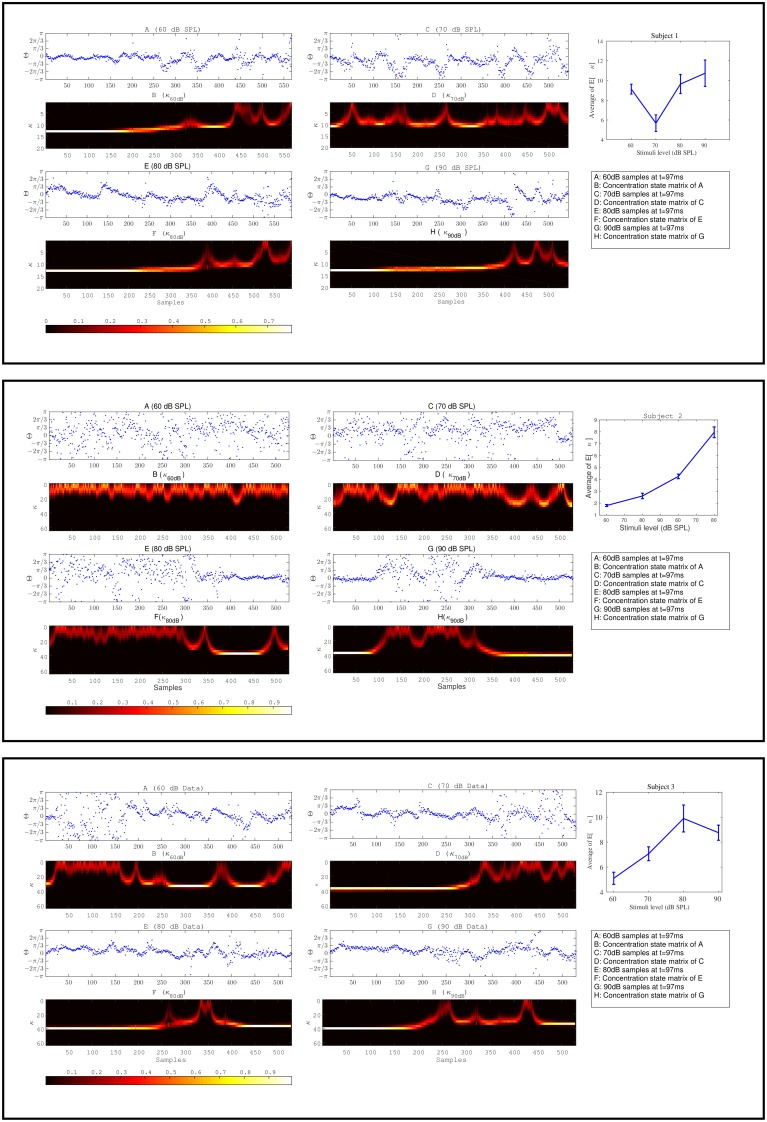
**The plots in box [1] and [2] and [3] correspond to the results of three different subjects**. Plots **(A–H)** for every box correspond to the forward-backward results of the concentration likelihood over trials at different stimuli. Part I is the expected value of concentration for data at different times *t*. Based on the magnitude of the expected value of the concentration parameter at different times (∀*st*) as shown in part I for every subject, we are able to distinguish between different stimuli levels.

The effect of habituation between different subjects is variable. For additional clarity, in Figure 11 we illustrate the expected value of concentration at different stimuli intensities along with the individual temporal changes in the concentration level for three different subjects. The individual results of three different subjects shown in Figure 11 show the change process in the concentration states of phase data over all trials at a specific time *t* = 97*ms* at different stimulus levels. The marginal likelihoods of the concentration parameter illustrate the detected changes in the phase synchronization. The results of 60 dB SPL in subject 1-(A,B) present a gradual diffusion in phase synchronization, whereas in 70 dB SPL (C,D), the degree of phase cluster on average is low and contains many cyclic changes. As in 80 dB SPL and 90 dB SPL (E,F) and (G,H), respectively, the phase clustering remains relatively high throughout the experiment before undergoing significant state transitions. This is particularly evident in case of 90 dB SPL in which a significant change occurs at *t* = 510*ms*. The averaged results of the concentration at different times suggest that the level of phase diffusion for higher stimuli levels such as 80 and 90 dB SPL are relatively lower. This indicates a stronger attention-binding to the stimulus because of the subjective unpleasant stimulus perception.

The same explanation applies to the other two subjects in Figure 11. We explain the findings as follows: The detected change process in the concentration states of 60 and 70 dB SPL in subject 2-(A,B) and (C,D) tend to fluctuate rapidly between the lower-concentration states. The average of the expected value of the concentration at these two stimuli are very low and contain little structural synchronization. The diffused phase information throughout the experiment indicate a very low attention-binding to the stimulus. In case of 80 dB SPL phase samples are uniformly distributed for the first 410 ms, followed by a higher degree synchronization, which is closely reflected in the fitted concentration states. In the last subject in Figure 11, a same type of explanation regarding phase modulations can be applied.

In Section 4 we show how to use the changes in the underlying estimated concentration parameter to objectively differentiate between different stimuli.

## 4. Discussion

The objective goal of this study is to evaluate the degree of habituation effect using the instantaneous phase of ERPs induced at different stimuli of 60, 70, 80, and 90 dB SPL. To do this, we used a Bayesian model to track the changes in the underlying concentration parameter of the instantaneous phase information of ERPs. In addition we used the verbal responses of the participants about the loudness of different stimuli. This knowledge was used to validate the conclusion about the relation between the objective measure and the loudness scale at different stimuli.

**Figure 12:**
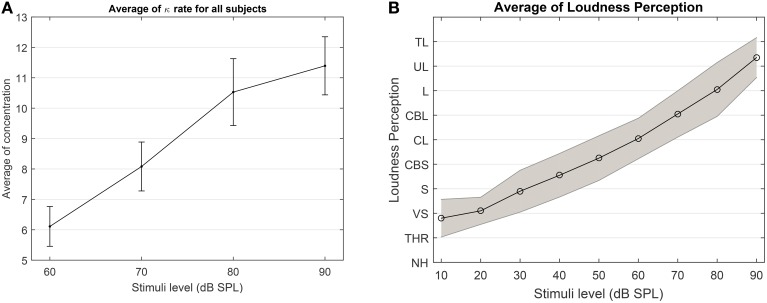
**(A)** The average expected concentration over all subjects at different stimuli levels. **(B)** The average over the loudness perception for all subjects at different stimuli. The loudness perception y-axis is defined as NH, Not heard; THR, Threshold; VS, very soft; S, Soft; CBS, comfortable but soft; CL, comfortable loud; L, loud; UL, upper level; TL, too loud.

Despite the high variability among subjects in terms of changes in the concentration states (see in Supplementary Figure 1), the average results of the concentration in Figure 12 suggests that *as the loudness level increases, it is highly probable that the degree of phase synchronization increases as well*. To validate the obtained results, we compare the averaged results of the level of concentration against the average of the verbal responses of the participants. As shown in Figure 12B, as stimuli level increases the intensity of the loudness perception increases as well. This is consistent with the studies conducted by Hood and Poole (1966) and Stephens and Anderson (1971), that the stimuli between 90 and 100 dB SPL are considered as uncomfortably loud in normal hearing subjects. Furthermore, we applied a one way ANOVA test over different stimulus levels across all subjects and it can reliably distinguish between the 60 and 90 dB SPL using the average of the expected concentration at a significance level of 5% with *p* = 0.0101(*F* = 7.37).

**Figure 13:**
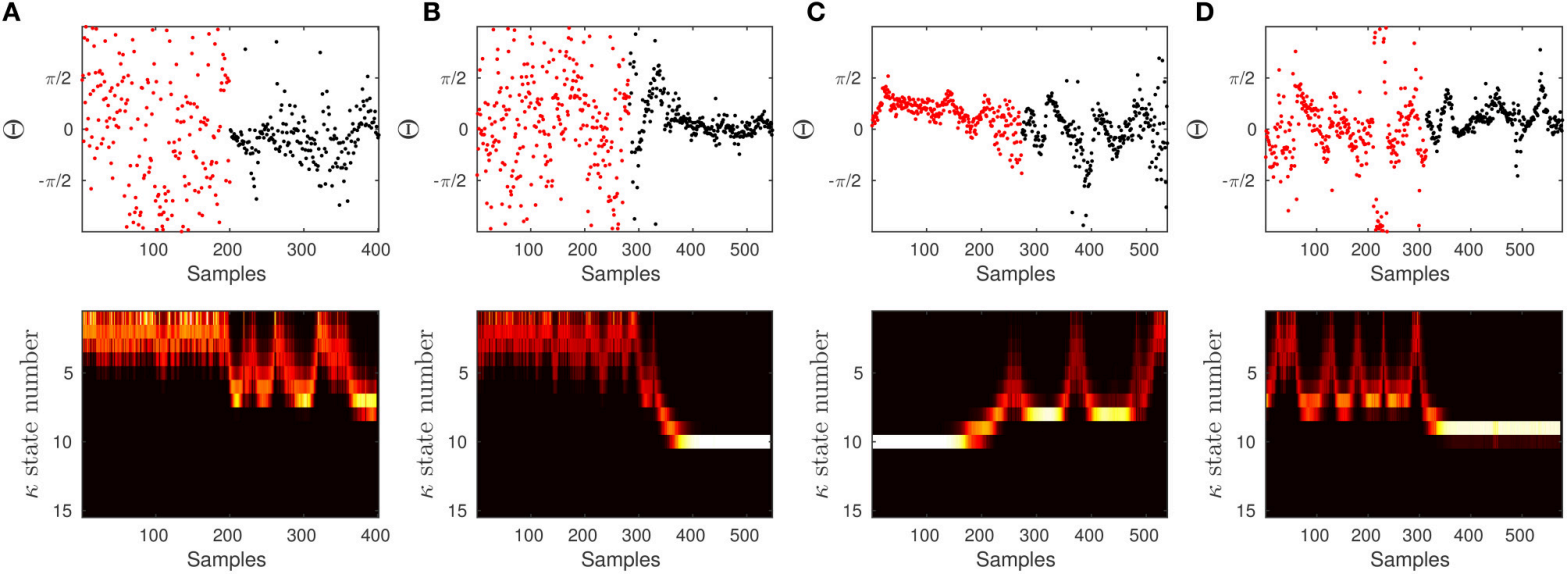
**To show the effectiveness of the algorithm to significantly differentiate between 60 and 90 dB SPL, we constructed a signal which is composed of two parts**. The first half of the signal (in red) corresponds to the first half of a signal at 60 dB SPL, and the other half (in black) corresponds to the second half of a signal at 90 dB SPL. We run the proposed algorithm on data with the empirical prior parameters and check if the model is able to track the distribution of data in terms of the concentration.

To test the effectiveness of the algorithm on experimental data and the significance of the results between 60 and 90 dB SPL, we applied an additional test as follows: we generated a signal which consists of two parts, the first part corresponds to the data samples from the first half of the 60 dB SPL and the second part of the signal contains the samples from the second half of the 90 dB SPL data. We applied the proposed forward-backward Bayesian model with the same empirical prior parameters as in Section 3.3 to check if we are able to detect the artificial change point between two stimuli. In Figure 13, we show a few examples of our observations. Throughout all observations, the model is able to track the distribution in terms of the changes in the concentration parameter.

In some of the tests it is more difficult to detect the artificial transition. This is mainly because the data in 60 and 90 dB SPL can behave the same at different time intervals. If the samples in the first half of the 60 dB SPL have lower concentration and the second half in 90 dB SPL is in a similar state, then the transition may not be evident. The same argument holds when different halves of the signals have high concentration values. However, given the results and the additional test, we can confidently report on the significant difference between 60 and 90 dB SPL.

### 4.1. The rate of change of the concentration parameter

To test whether the stimulus level has an impact on the *degree of change* in the concentration over trials, we conducted a second ANOVA test as follows: We adjusted the subject specific factor in the concentration values by dividing the expected values of the concentration by the average of the expected value of the concentration of the last 50 samples (the resulting values are denoted as **W** in Algorithm 1). The meaning of the resulting values can be understood as follows: In case of no change at all, all values in **W** will be exactly 1. If the concentration increases for a subject at a given stimulus level throughout the experiment, values at the beginning of the experiment will be < 1. A decreasing level of concentration corresponds to values >1. We next compute the average over the divided expected values of the concentration for every subject at different stimulus levels. This shows the relative changes in the phase concentration over trials with respect to the last 50 samples. A One-way ANOVA test yields the following results: The null hypothesis that the relative changes of the expected of concentration is independent of the stimulus level can be rejected at a significance level of 5% with *p* = 0.0178(*F* = 3.58).

### 4.2. Limitations

Despite the success to objectively differentiate between different stimuli by using the instantaneous phase information of the ERPs, and determine a significant difference between the rate of change in the concentration parameter among different groups, the variability in the dynamics of the instantaneous phase among subjects is very large. Therefore, it is difficult to draw additional general conclusions, such as determining a general time at which habituation may occur for different subjects, or to conclude a unique uncomfortable sound level threshold among all subjects. Our results are also constrained by the limited amount of data.

As an example of habituation variability, we describe the behavior of habituation effect for 60 and 70 dB SPL for subjects 1 and 2 in Figure 11. The corresponding results for subject 2 show that the phase information is uniformly distributed throughout the experiment and as a result, the estimated concentration fluctuates rapidly between the lower concentration states. However, the habituation process in subject 1 is visible as a continuous decay in the phase concentration. The change process in concentration is more significant in the first subject than the second one, however based on the average of the expected value of concentration, it can be concluded that in both cases a weak level of attention has been allocated to the stimulus due to low expected value of the concentration. This effect is mainly due to the variances of neural responses over the number of stimulus presentations. The same acoustic stimulus presented to a subject never elicits identical neural responses across a series of presentation. While this effect holds for a single subject (intraindividual variability), the effect is even more pronounced across a group of subjects (interindividual variability) due to physiological variations in the neural architecture. Moreover, our experimental paradigm can not force the participants to ignore a presented stimulus. The information carried by the stimulus is not limited to its intensity. Associative processes in the individual subject can alter the subjective impact of a stimulus (see Busse et al., 2009) and lead to a variation of time periods in which the subjects voluntarily pay attention to the presented stimulus. At this stage, with the current data and analysis tools, we believe it is not possible to describe a definite habituation onset in time which is solely determined by stimulus intensity.

### 4.3. Future work

There are several promising directions for future work:

- We can try to join similar repetitive state transitions into a single state and show only the state transitions with significant differences. This could be achieved by applying a discrete two state Hidden Markov Model on the results of the marginal value of the concentration parameter.
- To adapt the scale of the states with respect to the overall average of the expected value of the concentration (i.e., different resolutions). Applying such mechanism will help us to track the behavior of the habituation in cases that a weak attention level has been allocated to the stimulus.

Additionally, one other area which could improve our results is denoising the phase information more effectively as a pre-processing step. In this study the phase information was extracted after denoising the ERPs using a NLM algorithm. We suggest to utilize denoising methods directly on the phase data, to reduce the noise level in the phase information.

### Conflict of interest statement

The authors declare that the research was conducted in the absence of any commercial or financial relationships that could be construed as a potential conflict of interest.
