# Supplementary material for: Bayesian Modeling of the Dynamics of Phase Modulations and their Application to Auditory Event Related Potentials at Different Loudness Scales
Source: Front Comput Neurosci. 2016 Jan 28;10:2. doi: 10.3389/fncom.2016.00002 (PMC4730906; doi:10.3389/fncom.2016.00002)
Supplement: Supplementary file 1 [file Appendix.pdf]

APPENDIX

** The parameters of the algorithm are set as follows: **
** numStimuli = 4, refering to the different stimuli levels. **
** numSubjects = 20, refering to the subjects used in this study. **
** N, the number of trials per subject/stimulus level. **
** Matrix Initializations:* $\boldsymbol{W} \in \mathbb{R}^{numSub \times N}$, $\boldsymbol{A} \in \mathbb{R}^{numStimuli \times N}$, $\mathbf{V} \in \mathbb{R}^{\text{numStimuli} \times \text{N}}$.
** Vector Initializations:* $\boldsymbol{v}' \in \mathbb{R}^{numStimuli \times 1}$, $\tilde{\boldsymbol{v}} \in \mathbb{R}^{N \times 1}$.

for *each candidate configuration* (σ^2, K) **do**
  for *g=1:numStimuli* **do**
    for *i=1:numSubjects with data set* $\boldsymbol{\Theta}_{i,g}$ **do**
      for *t=1:N* **do**
        Compute $p(\kappa_t, \mu_t | \boldsymbol{\Theta}_{i,g}, \sigma^2, K)$ using the forward-backward algorithm.
        Marginalize over μ_t: $p(\kappa_t | \boldsymbol{\Theta}_{i,g}, \sigma^2, K)$;
        Compute the expected value $E[\kappa_t] = \sum_{\kappa_j \in \boldsymbol{\kappa}} \kappa_j p(\kappa_t = \kappa_j | \boldsymbol{\Theta}_{i,g}, \sigma^2, K)$;

        Store the result in the matrix $\mathbf{W}_g$.
        $\mathbf{W}_g(i,t) = \mathrm{E}[\kappa_t]$;
      end

      Adjust for subject-specific factor in concentration values:
      * divide κ by mean κ of the last 50 data points*
      $\mathbf{W}_g(i,:) = \mathbf{W}_g(i,:)/avg(\mathbf{W}_g(i,(N-50):N))$;
    end

    Compute the group mean and in-group variance at each time t
    for *t=1:N* **do**
      Store the group mean at time t, averaging over all subjects.
      $\mathbf{A}(g,t) = avg(\mathbf{W}_g(:,t))$;
      Store the in-group variance at time t.
      $\mathbf{V}(g,t) = variance(\mathbf{W}_g(:,t))$;
    end
    Average over in-group variances at different t: $\boldsymbol{v}'(g) = avg\left(\mathbf{V}(g,:)\right)$.
  end

  Compute between-groups variance, at each time t
  for *t=1:N* **do**
    $\tilde{\boldsymbol{v}}(t) = variance_g(\mathbf{A}(:,t)$
  end
  Average over between-group variances at different t: $\tilde{v}' = avg(\tilde{\boldsymbol{v}})$

  Compute the optimization criteria
  $\rho(\sigma^2, K) = \frac{\tilde{v}'}{avg_g(\boldsymbol{v}'(g))}$;
end

Algorithm 1: The algorithm used for computing the optimization criteria ρ for each set of prior parameter candidates (K_i, σ_j^2).
